# Exploring views of South African research ethics committees on pandemic preparedness and response during COVID-19

**DOI:** 10.1177/17470161241250274

**Published:** 2024-05-04

**Authors:** Theresa Burgess, Stuart Rennie, Keymanthri Moodley

**Affiliations:** Stellenbosch University, South Africa; University of Cape Town, South Africa; Stellenbosch University, South Africa; University of North Carolina, Chapel Hill, USA

**Keywords:** Research ethics committee, COVID-19, pandemic preparedness and response, public health emergency, ethics

## Abstract

South African research ethics committees (RECs) faced significant challenges during the COVID-19 pandemic. Research ethics committees needed to find a balance between careful consideration of scientific validity and ethical merit of protocols, and review with the urgency normally associated with public health emergency research. We aimed to explore the views of South African RECs on their pandemic preparedness and response during COVID-19. We conducted in-depth interviews with 21 participants from RECs that were actively involved in the review of COVID-19 related research, at seven academic institutions across South Africa. Interviews were conducted remotely using an in-depth interview guide that included questions regarding REC preparedness and response to COVID-19. Interviews were conducted until data saturation, and audio-recordings were transcribed verbatim and coded. An inductive approach to thematic analysis was used to organise data into themes and sub-themes. This study focused on three main themes: coping during COVID-19, building REC capacity during pandemic times and a consistently cautious approach to mutual recognition of REC reviews. Despite an initial sense of unpreparedness, RECs were able to adapt and maintain careful ethical oversight of both COVID and non-COVID research, and the rigour of REC reviews. Several important lessons for preparedness and response to future pandemics were identified, including heightened awareness of publication, funding and political pressures, the importance of regular training for RECs and researchers, and strategies to enhance moral resilience of REC members. Incremental steps are needed to build trust and authentic partnerships among RECs in inter-pandemic times, to facilitate collaboration during future public health emergencies.

## Background

The COVID-19 pandemic has presented unique challenges to global public health. Vulnerable populations have been disproportionately affected and existing health inequities have been exacerbated (Shadmi et al., 2020). Significant research efforts have informed COVID-19 disease pathogenesis and clinical management practices, vaccine development and rollout and public health policies and strategies (African Academy of Sciences, 2021; Kohrt et al., 2019; Mullard, 2020; O’Mathúna and Siriwardhana, 2017; Roope et al., 2021).

The critical role of research ethics committees (RECs), also known as research ethics boards (REBs) or institutional review boards (IRBs), in reviewing research during public health emergencies hhas been highlighted, particularly in countries with limited health infrastructure and resources (Aarons, 2019; Alirol et al., 2017; Schopper et al., 2017). Research ethics committees are often required to review innovative, but potentially high-risk, research to address public health emergencies. Novel or repurposed investigational products may be trialled in highly vulnerable patient populations with limited or newly available knowledge, as was experienced during the height of the COVID-19 pandemic, complicating determinations of risk-benefit ratios, scientific validity and social value (Salamanca-Buentello et al., 2023).

International guidelines for research during public health emergencies have stressed the importance of maintaining ethical principles for research during disease outbreaks (Council for International Organizations of Medical Sciences, 2017; Pan American Health Organization, 2016; World Health Organization, 2010, 2015, 2016) and the need for robust ethics review processes (Hunt et al., 2016; Saxena et al., 2019; Tansey et al., 2010).

The tensions that exist between upholding the rigour of research ethics review and the urgency to produce valuable scientific knowledge aimed at advancing prevention and treatment of public health emergencies have also been described (Alirol et al., 2017; Doroshow et al., 2020; London and Kimmelman, 2020; Schopper et al., 2017; Sigfrid et al., 2020). Research ethics committees are also tasked to determine the ethical merit of the research in the face of significant uncertainty and often in sub-optimal conditions (Salamanca-Buentello et al., 2023). During public health emergencies, RECs may also need to adjust processes and policies to improve the efficiency of the review process, and ongoing ethical oversight of approved protocols while still adhering to ethical principles and guidelines (Salamanca-Buentello et al., 2023; Tansey et al., 2010). However, although international guidelines provided valuable recommendations for the ethical review and oversight of research during the COVID-19 pandemic (Pan American Health Organization, 2020; World Health Organization, 2020b), there is limited information regarding the preparedness of research ethics committees in Africa to operationalise these guidelines, or how RECs responded to the COVID-19 pandemic.

In South Africa, all institutional-level RECs that review health research are required to register with the National Health Research Ethics Council (NHREC), in accordance with the National Health Act 61 of 2003 (National Department of Health, 2015; Republic of South Africa, 2003: Section 1.6.2; p. 11). There are currently 46 RECs that review health research involving human participants registered with the NHREC across South Africa (National Department of Health, 2022). The core functions of the NHREC include advising the Minister of Health to set ethical norms and standards for health research and to advance research ethics in South Africa by promoting the use of existing and new research ethics guidelines by RECs and researchers. The National Health Act requires that the Minister of Health appoints 15 NHREC members who have knowledge and experience in research ethics or the law, and who are committed to promoting research ethics. The NHREC is generally reconstituted every 3 years (National Department of Health, 2015; Republic of South Africa, 2003).

However, the National Department of Health did not reconstitute the NHREC from November 2019 to December 2020. This resulted in a critical absence of national-level research ethics guidance and oversight during the first and second waves of the COVID-19 pandemic in South Africa (Moodley, 2020; Rossouw et al., 2021). It is unclear how institutional-level RECs operationalised national (National Department of Health, 2015) and international guidelines for research during the COVID-19 pandemic (Pan American Health Organization, 2020; World Health Organization, 2020b). In addition, experiences of RECs in sub-Saharan Africa during the COVID-19 pandemic have not been widely reported in empirical ethics literature. Against this background, our research aimed to explore the views of South African RECs on their pandemic preparedness and responses during COVID-19.

## Methods

### Data collection

This study was reviewed and approved by the Health Research Ethics Committee at Stellenbosch University (N20/10/062_COVID-19) and the Faculty of Health Sciences Human Research Ethics Committee at the University of Cape Town (HREC REF 045/2021). Additional institutional or stakeholder permissions (such as permission to access staff employed by academic institutions) were also obtained as needed. Research ethics committee chairs and members who were actively involved in the review of low- to high-risk COVID-19 research were purposively sampled. We chose purposive sampling to identify and select information-rich participants who were knowledgeable about or experienced in the phenomenon of interest (Cresswell and Plano Clark, 2011), namely REC operations and functioning during the COVID-19 pandemic and ethics review of COVID-19 and non-COVID-19 research. All participants reported having reviewed a range of COVID-19 protocols that included observational studies, preventative and diagnostic studies, therapeutic clinical trials, vaccine trials and implementation studies. Twenty-one REC chairs and members consented to take part in this study.

Participants represented 10 different RECs across South Africa, including academic institutions (*n* = 7), national governmental organisations (*n* = 2) and a charitable organisation (*n* = 1). Further, the RECs represented in this study were also thought to be responsible for the ethical oversight of approximately 75% of all COVID-19 clinical trial research that took place in South Africa during the pandemic (NHREC, personal communication, 29 November 2021).

The data for this paper were obtained as part of a parent study that broadly explored ethical challenges experienced by South African RECs during the COVID-19 pandemic (Burgess et al., 2023). The full interview guide for the parent study included questions regarding both ethical challenges faced by RECs during COVID-19 and REC preparedness and response to COVID-19. In this paper, we are focussing specifically on data from responses to questions pertaining to REC preparedness and response to COVID-19. We also included specific questions regarding reciprocal review, also known as ethics review mutual recognition, given the importance of harmonising and expediting ethics review in the context of a public health emergency (Rahimzadeh et al., 2023). We used the term ‘reciprocal review’, as this is commonly understood in the South African REC context and is defined in the National Department of Health’s Ethics in Research Guidelines (National Department of Health, 2015) as the recognition by a REC of a prior review and approval of a research protocol by another REC to avoid duplication in effort. We did not collect any information, including details of studies under review or approved by the RECs, that might identify participants, investigators, or their respective institutions.

Interviews were conducted via Zoom by one researcher (TB) with training and experience in clinical research, research ethics and qualitative methods. All participants gave written informed consent prior to being interviewed. Participants were requested to select a venue that ensured privacy and confidentiality of the interview. Interviews were conducted in English using an in-depth interview guide, and the average interview duration was 75 minutes (range 60–125 minutes).

Interviews were conducted until data saturation was achieved (Fusch and Ness, 2015; Hennink et al., 2017). Field notes were generated about interviews by taking small, keyword-based notes during interviews while maintaining participation and eye contact with participants. Comprehensive field notes were created shortly after each interview and included critical reflection of details of the interview, including any changes to interview questions (Phillippi and Lauderdale, 2018) and for the researcher (TB) to assess their performance, biases and feelings (Phillippi and Lauderdale, 2018; Watt, 2007). We did not document nonverbal behaviours during each interview, as interviews were conducted remotely. Field notes were coded with the participant number and stored as a separate document to the interview transcript (Phillippi and Lauderdale, 2018). Interviews were recorded and transcribed verbatim by an experienced research assistant. Transcripts were coded and stored in a OneDrive folder that is only accessible to the researchers.

### Data analysis

Transcripts were reviewed for accuracy by one researcher (TB) and were imported into Atlas.ti (Version 6.2.28 Windows, ATLAS.ti Scientific Software Development GmbH, Berlin, Germany) for analysis. Two researchers (TB and an experienced research assistant) developed the codebook by independently coding four transcribed interviews. Coding and emerging themes were then collectively discussed with the full study team (TB, SR, KM) and refined until an acceptable level of consensus and inter-coder reliability was achieved (Morse et al., 2002). Two researchers (TB and an experienced research assistant) then coded all transcripts. An inductive approach to thematic analysis (Braun and Clarke, 2006; Vaismoradi et al., 2013) was used to analyse data. Data were organised into themes and sub-themes, and the full study team (TB, SR, KM) consulted regularly to review the analysis.

## Results

### Participant characteristics

Twenty-one interviews were conducted with REC chairs and members. All participants had undergone research ethics training in the last 3 years, in accordance with the requirements of Section 4.4.2(iv) of the South African National Department of Health’s Ethics in Research Guidelines that outlines the NHREC’s expectations of institutions and REC members. Although these guidelines do not provide details of the nature and scope of research ethics training required by REC members, the NHREC requires that training should occur on appointment, be refreshed at least once every 3 years, and be evidenced by a certificate (National Department of Health, 2015). Eleven participants also had formal postgraduate qualifications in bioethics (*n* = 2), applied ethics (*n* = 1), and research ethics (*n* = 8). One participant had experience in regulatory affairs. The majority of participants (*n* = 14) were experienced in professional care, counselling, or health-related care. Four participants were laboratory or medical scientists, and one participant was legally qualified. The descriptive characteristics of participants are shown in Table 1.

### Themes and sub-themes

Our qualitative analysis revealed three main themes related to preparedness and responsiveness of South African RECs to COVID-19 research. The themes and sub-themes are outlined in Table 2.

### Coping during COVID-19

Our first theme was identified as ‘Coping during COVID-19.’ Many participants highlighted the overall perception that South African RECs were initially unprepared for COVID-19. The lack of preparedness was compounded by both the impact of the national lockdown on research and the operational challenges associated with the national lockdown. The perception of unpreparedness varied across different institutions and seemed to be related to the nature of institutional research ethics support systems that were in place prior to the pandemic. For example, REC chairs and members from institutions that had implemented electronic submission processes prior to the national lockdown and COVID-19 pandemic seemed to be able to transition more easily to remote online working, whereas RECs that were utilising paper-based submission processes struggled to adapt and maintain REC administrative requirements:

*I think some [research ethics] committees have coped really badly, unfortunately. I think we are very, very poorly, poorly resourced. To give you an example, the REC doesn’t have an electronic submission system. Everything is submitted via email now* [during COVID-19], *but it’s a paper-based system. So, there’s no intelligence. We can’t follow up on annual approvals because we don’t know what has been approved in the last five years unless we go through the physical records. And they’ve* [the REC] *been promised electronic system now for years, but it’s just not been implemented. It’s as if it is just not a priority*.

(P14)

In addition, the scope and volume of COVID-19 and non-COVID-19 research overseen by the respective RECs during that period also impacted on the perception of unpreparedness. For example, chairs and members from RECs that did not review medium- to high-risk COVID-19 research protocols seemed to reflect more on their personal experiences during the COVID-19 pandemic and other changes in workload and responsibilities, rather than heightened uncertainty associated with rapid ethics review of clinical trial research during COVID-19. A participant from a REC that reviewed predominantly low- to medium-risk social sciences research commented:

*I think we’ve coped well, under the circumstances. A lot of our committee members, me included, had to deal with personal loss and grief. And professional loss too. We’ve lost a lot of colleagues during this time… there was also added pressure because of course of our REC members have their own research, their academic responsibilities, their students, and their colleagues. They had to be there for them, in addition to supporting the research community*.

(P21)

In contrast, a participant from a REC that reviewed high-risk COVID-19 clinical trials reflected:

*With COVID-19 research, there’s a pressure to get it reviewed, and then hopefully get it approved, together with a huge amount of uncertainty. If you take the example of the hydroxychloroquine, there was a little bit of biological plausibility and some early bits of data suggesting it may be a good idea* [as a therapeutic intervention for COVID-19]. *But as a REC, you’re working in that environment where your* [the REC] *decision might change, according to a manuscript that’s published on the preprint server, like the night before* [the REC meeting], *suggesting there’s more toxicity or there’s less toxicity. And that makes it a whole lot more difficult to be certain about, if one* [the REC] *should be approving the studies*.

(P8)

Most participants indicated that the strain associated with REC work increased significantly during COVID-19. At a very basic level, the strain of REC work was related to the increased workload of RECs during the COVID-19 pandemic:

*I find that’s probably part of the rapid review system that has been quite hard, is that for persons actually supporting research ethics committees, it’s been this additional layer of admin, that has suddenly erupted. So yeah, we need to just say that it’s* [COVID-19 rapid reviews and remote working] *all placed a really heavy burden on committees*.

(P4)

The strain of REC work was multi-faceted and complex and seemed to be magnified at the level of REC chairs and deputy chairs:

*If your REC does not have an online ethics administration system, it’s not been as easy to adapt [to working remotely]. We don’t have a robust online system and because of this, we’ve had to do everything from scratch. And I think that’s also put an extra burden on our admin team, as well as on the Exco* [REC Chairs and Deputy Chairs]. *In terms of burden to the committee members, they may have had to come to more meetings. We have asked some reviewers to assist in reviewing more studies, more quickly. But I think members have the option to opt in and opt out of attending meetings, which makes it easier to manage the strain. Reviewers can also decline to review protocols. Whereas the Exco* [REC Chairs and Deputy Chairs] *can’t opt out, and our admin team can’t opt out either. And so, I think the strain, the burnout, the fatigue is worse for the Exco* [REC Chairs and Deputy Chairs] *than for the committee members themselves*.

(P2)

*So, you do your regular work. And the research ethics chairing is just done on top of everything else. It’s not really recognised in any of the performance agreements or anything. We’re also very short staffed in terms of secretarial support. So, our one deputy chair takes responsibility for all the ethics admin. But now, with COVID-19, she also had to run the COVID-19 ward on her own some days. She’s been dragged into clinical care much more than usual. And she actually wanted to resign last month. She said, you know, I’m no longer coping. I can’t deal with all the correspondence [related to REC administration]*.

(P14)

Some participants also described the unanticipated and unprecedented pressures with initially being required to stop non-COVID-19 research, in line with the South African government’s national lockdown levels. The ethical complexities associated with stopping and then restarting non-COVID-19 research required increased REC oversight, as researchers were required to provide an ethical justification for restarting non-COVID-19 research. To restart, researchers were also required to provide standard operating procedures for conducting research during COVID-19 that included details of infection prevention and risk mitigation strategies, including social distancing. Research ethics committees needed to review all these standard operating procedures, risk mitigation strategies and protocol changes:

*So, RECs now had urgent, expedited applications* [for COVID-19 research], *but they also to manage stopping non-COVID research and amendments and* [protocol-specific] *SOPs to re-commence non-COVID research down the line. So, RECs were extremely overburdened in having to do not just one thing. The other thing that they struggled with, that I saw, is making mind switches between what would be the guidance for non-COVID research*… *and COVID-related research*.

(P18)

*Some of the researchers were very forthcoming in sending us an email and telling us, look, I feel really frustrated with this* [stopping of non-COVID-19 research]. *I have fears for when my student can actually start research*… *And [when remote online research was permissible] some researchers said, you can’t tell me, I can’t change my research process to do online research, I have to do it this way. So, it was a negotiation process, where RECs had to explain to the researchers the rationale behind restricting research contact* [during COVID-19]… *So, there was a lot of consultation and helping researchers plan and amend their research. But we [the REC] got some very fierce criticism, where researchers felt that the REC does not have the right to put restrictions on research. And that their criticism was in terms of academic freedom*… *that RECs were adopting a very paternalistic stance in enforcing that this is how researchers should navigate conducting research during the pandemic*… *and that this was not their* [the REC’s] *mandate*.

(P21)

The strain associated with the increased workload of RECs was compounded by operational challenges associated with both working remotely and needing to adapt administrative processes and functioning, but also when REC work was identified as an essential service, bringing ethics administrators back to work safely, with appropriate infection control and risk mitigation strategies. No one reported that RECs received additional resources during this period:

*I think administrative structures within the institution have been found out a little bit by the pandemic. There’s been no additional resources at all. Despite the fact we’ve made every effort to ask for support and we’ve had many meetings around that, there’s been no additional resources. And that could be because the institution itself is completely swamped by needing to build up their own policies… and were also slow to identify challenges with both working remotely and bringing people back to work safely and in a supportive manner, with good risk mitigation and infection control… with [institutional leadership] seeming to not realise that research ethics administrative structures, systems, and support are quite critical, actually*.

(P1)

*I think the admin [REC administrators] struggled greatly, and they’re still struggling greatly, I think this is because they’ve been off site, and they don’t have proper processes or resources. And we’ve been subject to some aspects where there’s been poor administration because we are so familiar with picking up issues easily when you are face-to-face and around all the time, which is harder to do when working remotely*.

(P3)

The general urgency and uncertainty associated with the COVID-19 pandemic also contributed to the strain of REC work. A common issue that was highlighted was the concern for reviewer fatigue with the volume of COVID-19 research requiring review, the rapid turnaround times for reviews, the complexities of reviewing COVID-19 research when science was rapidly evolving, and the high levels of uncertainty, particularly with evaluating potential risks and benefits for research participants. Most participants reported the perception of substantial political and social pressure to rapidly approve COVID-19 research, particularly with the shift towards trialling vaccine candidates:

*… And we maybe felt the pressure but didn’t succumb to the pressure of the investigators or whoever wanted us to do this quickly without reviewing properly*.

(P18)

*The problem with the fatigue of the* [research ethics] *committee is that they you have to rely on people being energised and not just being flippant to the process, because coming to the meeting and saying well, we do due diligence, but in fact, not reading the documents and not looking critically at the risk and benefits and it’s critical for this disorder because we know nothing about it*.

(P1)

Among REC chairs, it was commonly perceived that the absence of the NHREC during the initial waves of COVID-19 and national lockdowns was highly problematic. The lack of national-level guidance or policies to assist RECs in navigating the complexities of research during the COVID-19 pandemic was noticeable and limited the promotion of an institutional culture of ethical research and strategies to enhance scientific rigour and integrity (Lipworth et al., 2023). In addition, some REC chairs commented that the failure to reconstitute the NHREC during the critical period when the research landscape was rapidly changing signalled disinterest and was a missed opportunity to highlight both the important role of RECs in protecting research participants and in maintaining ethical oversight for research:

*I think a gap in leadership is always deeply problematic*… *The failure of the* [South African health] *ministry to appoint the NHREC signalled a disinterest in the ethical standards of research? So, I think that’s deeply problematic in terms of fostering a culture of accountability, and a culture of ethical research. If, the NHREC is not there, then why should anyone else care? To disrespect legal obligation, and to fail to provide the highest level of leadership, then why should institutions care? So, at that level, the absence of the NHREC was deeply problematic*.

(P10)

*Procedurally, it was a feeling of discomfort… so if there is a problem, there isn’t anyone to fall back on*.

(P9)

*Not have a National Research Ethics Council available to us in a pandemic is in fact is negligence… it is really unacceptable*.

(P15)

Most participants also recognised the interconnectedness of complex financial, social and political pressures associated with COVID-19 and national lockdowns in the South African context:

*But what they’re not doing is actually working in the COVID space themselves. And what that means is that they’re purely publication driven or financially driven in terms of the grants, because a lot like in the HIV era, lots of money was being pumped into the COVID space to try and elucidate what is going on… And that I have noticed there’s a difference between trying to understand what is actually going on and do maybe better research, rather than opportunistic research within the COVID environment*.

(P1)

*Obviously, institutions will definitely want to be seen to be doing COVID-19 research… And then funders, because altruistically or non-altruistically, you want to be funding COVID-19 research. And the government, research is good politically… So, the [COVID-19 research] space opens up, and opportunities open up and sometimes the participants and their families are forgotten about in these environments*.

(P2)

*I think COVID is probably an opportunity for certain research careers to really be set into orbit where other people’s research careers take a dive, with a lot of people that haven’t been able to meet the commitments of their grants. And yet, they’ve still had to support students and salaries and things like that, but they haven’t been getting work done. They haven’t been producing outputs. It’s disastrous for some people. So*, [these are] *some of the unintended consequences of COVID and prioritisation of COVID research*.

(P7)

Funding, publication and political pressures were identified, leading some participants to consider whether REC deliberations were sufficiently sensitive to these broad pressures associated with COVID-19 research prioritisation during the height of the pandemic:

*I think trust works in multiple ways. Researchers need to trust an ethics committee not to share all details of the research that is proposed, because research is an interesting endeavour, and it’s highly competitive. Funders need to trust in the ethics committee to do a good job because their reputations are on the line if they fund research and it’s going to be unethical. Ethics is also about shades of grey and to say clearly and unambiguously what is ethical and what is absolutely categorically unethical is often not that easy. We deal with subtle questions, where the answer of whether something is acceptable or not depends on the context. It depends on the people, it depends on the intention of the researchers, you know, are they credible? Do you believe that they have good intentions? Or do you believe that they will take shortcuts and or they are not trustworthy?*

(P10)

*And then I start wondering how much the local PIs are really just in it for their own their own career purposes, as opposed to actually improving the situation for the for the community. And these other potential motivations for doing the research are not clear on protocols submitted for review*.

(P9)

### Building REC capacity during pandemic times

We described our second theme as ‘Building REC capacity during pandemic times.’ Many participants viewed their involvement in REC meetings and reviews as their contribution to efforts to fight COVID-19. A strong sense of making a meaningful contribution associated with REC work was reported:

*The* [COVID-19] *pandemic has maybe stimulated a lot of people to be present*… *I don’t want to use the word solidarity, but that’s the word that I think fits best*… *that people feel that they may be contributing to something bigger than themselves and it’s one of the ways that many of the people on the committee can actually make a contribution*.

(P17)

*Being a REC member and attending the rapid review meetings, I found it was a privilege to be part of that. And to be able to contribute something that was addressing the pandemic. I’m not a medical doctor I can’t save lives in that way. But through my* [REC] *experience and expertise, I felt I was genuinely able to assist other researchers and review their protocols. And I wouldn’t say enjoyment was the right word, but I felt a sense of contribution. And that was a good feeling. And it was incredibly intense and time consuming. Not only to review the protocols before but also to attend more meetings, but I also felt that we became closer as a research ethics committee. And it was, I think, in a way a positive experience*.

(P6)

Some participants struggled with their dual roles as REC members and frontline healthcare workers, particularly as their frontline clinical work during the early stages of the pandemic was fraught with fear, desperation and uncertainty, and emphasised the urgency of effective therapy or prevention. Participants also recognised the unique circumstances of experiencing a public health emergency first-hand and frequently encountering COVID-19-related deaths or severe illness in their professional, community, or personal networks. Participants identified inherent conflicts of interests associated with REC work during the COVID-19 pandemic because of their clinical and personal experiences and clinical involvement, but they also reported being even more careful to ensure due diligence in reviewing and approving research protocols because of these inherent conflicts. However, many participants also recognised the moral distress associated with their conflicting clinical roles and personal experiences, together with the significant responsibilities associated with reviewing and approving COVID-19 research in desperate and uncertain times. Moral distress may result from lived experiences of traumatic or very stressful events, such as the COVID-19 pandemic, and may be experienced where moral actions or values are constrained (Norman et al., 2021; Williams et al., 2020). The extracts below reflect the duality of roles, the associated inherent conflicts of interest and the potential for moral distress:

*I think people* [REC members] *working in the clinical space, have availed themselves, because they wanted the research to be done. Everybody wanted the research to be done. Everybody saw the significance and the importance of the research that needs to be done. So, I think everybody tried their best to make sure that we’re not stopping good research from being done in the clinical spaces*.

(P5)

*If you’re a clinician treating the COVID patients yourself, wanting to get that COVID study off the ground, versus yourself as a REC member having to stand back and say, let’s take a slightly more thorough look at this research. As a clinician, you can bring the understanding of clinical practice and need, and what’s happening on the ground, at the frontline. But making sure you can step back [as a REC member] and check scientific rigour and ethics, this is essential, so we do good research*.

(P8)

*I think we* [REC members] *all have conflicts of interest. Fundamentally, I think we have a conflict of interest because we’re dealing with institution-related research. At a higher level, we are wanting to maintain a certain status as a research-led institution. I am sure our inherent biases or conflicts relate to our clinical area of expertise. So* [as an example], *whenever paediatric research comes up, well, I’ll think it’s quite important to do*.

(P9)

*I think there have been many pressures*… *There’s the public and political pressure of urgency to find an effective treatment or vaccine for COVID-19. There’s the pressure that the institution and researchers are wanting things* [REC reviews and approvals] *very quickly. And so, there’s a huge feeling that one needs to get on with it and move. I think the other pressure has been the massive expectation from investigators. Regular SMS’s, regular WhatsApp’s, regular phone calls to find out where things are in the review process, and the inundation of emails and messages of why things* [REC reviews and approvals] *haven’t happened, not only in the COVID-19 space, but in the non-COVID-19 research space too. So, I think the stress has been quite great, on top of the general pressures and uncertainties and fears from being in the midst of a pandemic, with so many unknowns and such rapidly changing science, all of which make the research ethics review process more complex*.

(P1)

While REC work during the COVID-19 pandemic was characterised by many challenges and pressures, a consistent sub-theme was that all participants demonstrated strong commitment to maintaining a high level of functioning during the COVID-19 pandemic and national lockdown:

*The general attitude and commitment of all involved* [REC members] *was really great*…*it stands out for me as the most important thing is how people* [REC members] *were trying to what they have to do in the best way possible*.

(P4)

*… The engagements have been really positive. There seems to be a lot of energy. A lot of interest around talking about COVID-19 research and we’ve actually seen that flow over into our regular committee meetings where there’s been a lot of good debate and enthusiasm for what the REC is meant to do*.

(P2)

Participants also commented on the challenges associated with online REC meetings, particularly the effort needed to maintain rigour in research ethics discourse and full engagement of the committee in deliberations around protocols for research ethics review to ensure all views were heard and that the REC’s decision-making was appropriately informed. While these pressures potentially challenged the rigour of research ethics reviews, most participants reported that RECs were able to maintain their due diligence and review standards for COVID-19 research:

*I think we didn’t compromise… despite the fact that there’s a pandemic and we try to be quick, but to not compromise in ethical principles that we that we subscribe to*.

(P16)

*So, we may have done everything quicker, or expedited things in a way that we could rapidly review, rapidly meet, rapidly get matters, rapidly give feedback. But what we did was exactly what we usually do. In fact, we probably were a bit stricter in terms of protection of participants, and asked investigators to be strict in themselves in terms of what they put in front of us*.

(P1)

*So, it’s tension between on the one hand having to present a united front in credible decision making… then, on the other hand, it’s the power of personal intuitions, and moral intuitions, and the power dynamics in a committee, meaning that some people’s moral persuasions or intuitions are articulated more forcefully and impact on decision making in ways that in other committees about dynamic might be different. So, decisions can be made in different ways, and it is so important to hear everyone’s voices in REC deliberations*.

(P10)

Innovative practices were adopted to facilitate the review of COVID-19 research and to maintain the rigour of research ethics review during COVID-19. Participants also described the importance of RECs being adaptive, agile and responsive, particularly as the COVID-19 evidence base developed so quickly. Some RECs collaborated with researchers so that they could be informed of ‘pipeline’ research protocols and anticipated dates of submission of protocols to RECs for review to optimise the availability of reviewers and to reduce the review burden on committee members. Participants reported being challenged to remain up to date with the latest research findings, and the importance of expert reviewers was highlighted. In addition, RECs were required to regularly review protocol approvals and to potentially revise their decisions and approvals based on updated evidence. Examples of early clinical trials where REC decisions for continued approval may need to have been revisited include hydroxychloroquine (Gautret et al., 2020) and convalescent plasma (Sullivan and Roback, 2020). One participant reflected on innovative REC practices as follows:

*That’s been my sense throughout this experience of the* [COVID-19] *pandemic is that there’s been the need to find solutions despite the discomfort and the challenges. But there’s also been amazingly good* [research ethics] *work as well, and positive spin offs*.

(P3)

Many participants highlighted the critical role of REC chairs in the COVID-19 research ethics discourse at the micro-level within RECs, at the meso-level between institutional RECs, and at a macro-level in engaging nationally and internationally with governmental advisory committees, regulators and research communities. Further, the absence of national-level research ethics governance for a substantial period of the pandemic stimulated engagement and collaboration between RECs. The early consultation between REC chairs led to the collaborative establishment of a national ad hoc group of REC chairs and members, Research Ethics Support in COVID Pandemic (RESCOP).

RESCOP was conceptualised by two REC chairs in early March 2020, and within a few weeks the RESCOP network had expanded to include over 80 REC chairs, members, and administrators. The main purpose of RESCOP was to provide a collaborative network of support for RECs, to develop guidance to strengthen the ethical review of COVID-19 research, and to provide a repository of resources for research ethics during public health emergencies and COVID-19. RESCOP also developed guidelines for rapid full-committee reviews of COVID-19 research in South Africa. The REC chairs consulted regularly on an ad hoc basis during COVID-19 regarding multi-site protocols submitted to their respective RECs for review and other research ethics challenges.

RESCOP also provided a platform for sharing pandemic ethics resources and information using an open-access Dropbox folder and a group email list to facilitate South African REC stakeholders’ access to topical COVID-19 research ethics resources and discussions (Rossouw et al., 2021) and was viewed in a consistently positive light by participants:

*I think one of the wonderful collaborations that did come up with was RESCOP, which I think has worked very well. It hasn’t been explored to its full extent, mostly because people are overwhelmed and tired, and they just don’t have the time*.

(P14)

… *I think one would have to strive for a more intense, slightly different paradigm*… *To some extent with COVID-19 and RESCOP opening up the dialogue between committees, where the different chairs are talking to each other about the various vaccines and* [COVID-19] *studies that are coming through and trying to harmonise, at least on certain issues*.

(P7)

### Consistently cautious approach to reciprocal review

Our third theme was described as ‘Consistently cautious approach to reciprocal review.’ When considering the potential for reciprocal review, also known as ethics review mutual recognition, the majority of participants expressed caution. There were overarching concerns regarding review consistency and competency across different institutional-level RECs. Many participants highlighted that RECs had different levels of expertise and experience and that this would impact on the overall standard or rigour of ethical review of protocols. There was also a concern that reciprocal review could introduce the potential for “ethics shopping” (P1), particularly due to the urgency associated with COVID-19 research prioritisation, whereby researchers could submit their protocols to a REC that may have less experience in reviewing high-risk research, that may have a reputation for less rigorous review, or that may just generally be inclined to give favourable reviews, and that these considerations may potentially result in the ability to bypass careful scrutiny of protocols:

*In South Africa, we have that very real situation where not all research ethics committees have the same level of expertise*.

(P21)

*But let’s face it, not all RECs, and their capacity and experience or reviews, are equal. The problem is the lack of consistency of review processes, and there does seem to be very different standards of review and requirements of review*.

(P9)

It was recognised that differential standards of review were particularly problematic in the context of COVID-19, where rapid reviews by different RECs of the same research protocol frequently raised different ethical concerns, potentially resulting in confusion and frustration to researchers. In contrast, a few participants voiced the potential benefits of wider, harmonised review and proposed that harmonised review would close possible gaps in review processes that might be missed by an individual REC review process. This was accentuated during the pandemic, as many participants identified that they found it difficult to keep up with the rapidly expanding science of COVID-19 and expressed concern that ethical issues related to the scientific validity of protocols may have been missed:

*I think COVID accentuates it* [differential standards of REC review], *because there’s so much more opportunity for things to slip through because of the extra pressures and the fact that it’s a public health emergency. And so maybe the deficiencies in reciprocal review that I think are there generally, might become amplified during COVID-19*.

(P7)

*Are all ethics committees competent, and are we having differential processes and causing confusion within the pandemic* [research] *space?*

(P1)

…*That’s what what’s making the reciprocal review difficult as well* [during the COVID-19 pandemic], *because if there are some critical issues that* [research ethics] *committees don’t agree on*.

(P20)

*I think everybody* [RECs and researchers] *might actually be able to benefit from getting a wider* [research ethics] *review, and fewer things* [ethical or scientific concerns] *would slip through the cracks*.

(P6)

Participants also identified the importance of the historical and political context. In the South African context, the exploitation and human rights abuses that are embedded within the country’s history (Moll, 2021; Newman et al., 2011; Tangwa and Munung, 2020; Thabethe et al., 2018) were seen as critical factors in support of the need to maintain the independence of RECs. Some participants considered that these historical and political contextual factors may also contribute to mistrust between institutions, and subsequently RECs, leading to hesitancy towards reciprocal review and poor uptake and acceptance of opportunities for reciprocal review:

*And you have to turn the clock back 20 years*… *There are still so many of the overarching issues that we have to deal with, before we can get to the more nuanced issues* [of reciprocal review]. *We are still dealing with aggressions. These are alive and well in our university and we have to deal with it. To be honest, we think we might be progressive sometimes, but then you often you get pulled back to the very overarching, very historical issues time and time again. We haven’t moved as far, if at all, forward as we would have liked to, and I think that because of our history of exploitation, because of our history of being abused, it [reciprocal review] is such an important issue. As research ethics committees, we have a collective responsibility to then get the NHREC or national government, if the NHREC is not reconstituted, to put a reciprocal review system in place*.

(P15)

*I don’t know if or how that word “colonialisation” applies here, to reciprocal review. I think there are developed countries and there are developing countries; there are rich countries, and there are poor countries, there are first world type countries who are less inclined to trust other countries, partly because of their perceptions of possibly inferior systems, or possibly due to lesser resources. I don’t know that people* [RECs] *in America or the UK particularly think of the* [historical] *colonies as being inferior. I don’t know that they even think of us in those terms. Maybe historically, but in today’s world, it’s perhaps more just about capacity and resources and trust. Possibly, a bit of arrogance, but I wouldn’t call it colonialisation*.

(P7)

There was a strong recognition for the importance of trust in building towards mutual recognition of REC reviews. The concept of trust was multi-faceted. Participants highlighted the importance of preserving and continually developing trust with the communities that they serve and protect. Trust also needed to be established between RECs to facilitate mutual recognition of reviews:

*I think a lot of the difficulty [with establishing a reciprocal review process] is that people don’t know one another. They’ve never worked together. And they just don’t trust the processes of the other RECs because they’re not transparent. It’s very difficult to find SOPs, who the [REC] members are and how they [other RECs] actually function? You don’t know the people on their committee. And I think that mistrust is leading into people not wanting to do reciprocal review. I think we have to move towards trusting other RECs, because this lack of trust delays research significantly*.

(P14)

…*It’s* [reciprocal review] *about trust and experience*… *if you did work with another ethics committee, and you became very familiar with their processes and reviewers*… *At the end of the day, you realise that you’re actually coming out on the same page* [with research ethics reviews], *then maybe you can move into some sort of reciprocal arrangement. I’d be happy with that*.

(P15)

*That’s the tension in reciprocity for me, being able to really look at whether there a need for oversight from the local* [research ethics] *committee and in the interest of the local participants, because I think we all work hard at sustaining a level of trust with our local communities*.

(P3)

Part of establishing trust included understanding REC competence and experience and working together to build capacity among RECs. Clear lines of accountability, in terms of which REC would serve as the committee of record, were also essential to ensure appropriate ethical and legal oversight of research:

*Who* [which REC] *is then ethically, legally, responsible for the outcome of the ethics review? Who would take that responsibility, where does it lie? We take us our responsibility seriously of being the final call when it comes to reviewing and approving a project*… *that will require some careful thinking*.

(P14)

Some participants also suggested that reciprocal review may also be facilitated by geographical proximity, particularly in South Africa, where there is wide diversity:

…*The closer* [geographically] *the other institution is to you, probably the more there might be in common* [between RECs], *and therefore potential for reciprocal review of certain things [types of research] might be acceptable. You also know that they are different [institutional] cultures*… *and have different nuances. And so sometimes what one institution wants is not necessarily based on ethics, but due to institutional culture*.

(P13)

In addition, participants also emphasised the need to promote community building and knowledge exchange between RECs to facilitate reciprocal review. Participants recognised the importance of creating a safe, inclusive community of practice for REC chairs and members. Capacity development, training and collaboration were identified as essential components of a community of practice:

*So, part of research ethics and being able to discuss the issues comes from understanding the* [scientific] *topics, in addition to the ethics*… *You teach the new people how things occur and what principles exist and what arguments can be made*. *… It’s important from a succession point of view, to have a constant adding and leaving of new and old blood* [to REC membership]. *But I think experience is critical. Without that sort of solid core, I don’t think the REC can function effectively. There is also an institutional memory, there is a certain gumption and commitment that you need to be a REC member, and even more so a REC chair. It takes years to actually get those kinds of skills, I think*.

(P18)

*Community is the answer, and a trusted community. And an environment where people can share information, questions and show their vulnerability, the fact that they don’t have all the answers, and seek advice from others*.

(P10)

## Discussion

Research ethics committees faced significant challenges during COVID-19, particularly during the early stages of the pandemic. South African RECs were initially unprepared for COVID-19 research but were then able to effectively pivot to rigorous rapid REC review processes, while absorbing substantial and diverse pressures and high workloads. Our findings align with results from studies in other low- and middle-income countries (LMICs; Canario Guzmán et al., 2022; Hinga et al., 2022; Joshi et al., 2023; Kadam et al., 2022; Marzouk et al., 2021; Palmero et al., 2021; Rossouw et al., 2021; Sisa et al., 2021) including high-income countries (Faust et al., 2021; Ford et al., 2021; Ijkema et al., 2021; Sisk et al., 2022; Taylor et al., 2021), and a recent international study (Salamanca-Buentello et al., 2023).

Importantly, we identified an awareness among RECs that COVID-19 research prioritisation was also associated with significant funding, publication and political pressures. To contextualise publication pressures during COVID-19, a 2021 study reported a 3% annual growth rate for biomedical and health sciences research outputs from 2009 to 2019, but this increased to an average of 16% in 2020 (National Science Board, 2021).

There was also an upsurge in both public and professional interest in scientific literature as the global community tried to learn more about COVID-19. Scientific literature also had a major impact on COVID-19 vaccine development, health policy and public health guidance (Taros et al., 2023). However, Retraction Watch (accessed on 29 February 2024) currently lists 404 retracted papers related to COVID-19, although the site does not distinguish between articles that have been formally retracted or withdrawn. A further 18 COVID-19-related articles are listed as having associated expressions of concern (Marcus and Oransky, 2020). Retracted publications frequently continue to be cited post-retraction, which means that incorrect or incomplete information about COVID-19 may continue to be disseminated, potentially resulting in future errors (Dinis-Oliveira, 2020; Taros et al., 2023).

An important lesson to be learned from COVID-19 is that RECs should have heightened ethical awareness to significant funding, publication and political pressures associated with public health emergency research. While it may be difficult to formally factor these pressures into research ethics review processes, a possible suggestion is that RECs should proactively engage with governmental advisory committees to enhance their understanding of political pressures, and with industry research and development (R&D) to keep abreast with new opportunities for innovation, research trends and market needs during public health emergencies. Research ethics committees should also be mindful of the potential ethical issues relating to scientific validity, ethical merit and risk-to-benefit ratio that may arise from premature or expedited publications. Research ethics committees should also consider formalising regular engagement with leaders in respective clinical specialisations, such as infectious diseases or immunology, public health and health systems and policies, to ensure that they remain informed and up to date on emerging evidence and the potential impact on REC reviews and approvals.

During public health emergencies like COVID-19 where science is rapidly evolving, RECs need to actively engage with evidence to identify any potential changes in equipoise, risk, benefit, or safety of approved studies. This is essential to facilitate informed decisions to suspend or terminate studies that were previously approved by RECs. We also recognise that, while these funding, publication and political factors may be more prominent during public health emergencies, RECs should consider these factors during inter-pandemic times. An important consideration is to continually review the membership composition of RECs to ensure appropriate capacity and expertise for research ethics reviews. In addition, we recommend that pandemic urgency should not offset the need for regular training in research integrity and the responsible conduct of research for all levels of researchers and REC members.

We observed that participants struggled with their dual roles as REC members and frontline healthcare workers, as well as frequently encountering COVID-19-related deaths or severe illness in their professional, community, or personal networks. Reviewer fatigue was also a concern. Some REC chairs reported a heightened sense of responsibility and associated moral distress during COVID-19. Research ethics oversight was an essential service during COVID-19 but was compounded by well-publicised global mortality rates, socioeconomic burdens and pervasive uncertainty (Barugahare et al., 2020). Within the South African context, there was also an acute awareness that COVID-19 disproportionately affected disadvantaged or vulnerable groups (Palmero et al., 2021; Shadmi et al., 2020; Tangwa and Munung, 2020; Wright, 2020) and that government restrictions to prevent infection limited access to basic health needs (Poole et al., 2020; World Health Organization, 2020a). The prioritisation of COVID-19 research negatively impacted on other infectious disease research, such as HIV and TB (Makoni, 2020; Velavan and Meyer, 2020), non-COVID-19 clinical research (Audisio et al., 2022), and non-clinical research (Raynaud et al., 2021). Research ethics committees were challenged to consider basic human rights, public health concerns, resource constraints and structural inequalities in addition to fundamental research ethics principles during COVID-19 (Lorenc et al., 2023; Shadmi et al., 2020).

We also identified elevated levels of stress and fatigue experienced by participants associated with the myriad of complex ethical issues and dilemmas requiring REC deliberation and oversight during the COVID-19 pandemic. Participants in this study reported moral conflict associated with their dual roles as frontline healthcare workers (HCWs) and REC membership. There was also heightened moral distress associated with the responsibilities and pressures of needing to review and approve COVID-19 research in desperate and uncertain times, particularly in the context of significant health inequities.

While moral distress among healthcare workers during COVID-19 has been well-documented (Al Maqbali et al., 2021; Amanullah and Ramesh Shankar, 2020; Lai et al., 2020; Morgantini et al., 2020; Pappa et al., 2020), we could not identify any literature that focussed on moral distress in REC chairs or members. We also noted that survey respondents in an international survey of research ethics review during COVID-19 suggested that it would be valuable to explore emotional and psychological challenges experienced by REC members during times of increased pressure and uncertainty, such as the COVID-19 pandemic (Salamanca-Buentello et al., 2023). As many participants in our study were HCWs, we felt that it was appropriate to draw on literature that has described moral distress in HCWs, particularly during the COVID-19 pandemic. According to Spilg et al. (2022), “moral distress arises when HCWs face moral adversity, must make a moral judgement about the most ethically justified response, and act on it in a situation where the consequences of action (or inaction) imperil their moral integrity” (Spilg et al., 2022: 1). Moral distress may result from lived experiences of traumatic or very stressful events, such as the COVID-19 pandemic, and are experienced where moral actions or values are constrained (Norman et al., 2021; Williams et al., 2020). Although moral distress may be transient and self-limiting, it may develop into moral injury (Čartolovni et al., 2021; Epstein and Hamric, 2009; Williams et al., 2020), which is characterised by degradation of individual health and well-being, including burnout (Norman et al., 2021; Morgantini et al., 2020; Spilg et al., 2022).

Factors reported to contribute to moral distress during COVID-19 included poor organisational support, increased workload, time pressure and high job-related stress (Morgantini et al., 2020) and ethical dilemmas (Donkers et al., 2021; Hegarty et al., 2022; Nagle et al., 2023; Silverman et al., 2021). Higher moral resilience has been associated with greater support from employers and colleagues. Studies have suggested that strategies to enhance individual moral resilience within the context of organisations may include strengthening relational integrity, working towards a shared moral endeavour, creating spaces for dialogue and discussion, and providing structured debriefing about difficult cases (Rushton, 2018; Spilg et al., 2022; Wocial et al., 2010).

Further, organisational strategies should focus on targeting potentially modifiable factors, such as the provision of additional training, organisational support and resources to offset increased workload demands during COVID-19 (Morgantini et al., 2020). A notable observation by Salamanca-Buentello et al. (2023) was that, despite massive investment into the research and development ecosystem to support COVID-19 research, little to no additional resources were directed to RECs to support their critical functions during the pandemic and may have contributed to the pressures experienced by RECs during COVID-19 (Salamanca-Buentello et al., 2023). Appropriate infrastructure for RECs to operate effectively and to appropriately facilitate ethically conducted research is essential. A key recommendation to ensure sustainable and improved REC functioning, both during and between pandemics, is the allocation of dedicated funding for RECs, as a set percentage of research funding. Research ethics committees also need to move towards more sophisticated, online ethics administration systems to support ethical oversight and monitoring of research. Institutional culture also needs to be explored to establish how the workload and skills of RECs are recognised and valued (Wright et al., 2023).

In South Africa, a key network of support for RECs was stimulated by the lack of national-level research ethics governance and oversight during the first and second waves of the COVID-19 pandemic. The absence of the NHREC during these times of heightened uncertainty was identified as a critical gap, and institutional-level REC chairs collaborated and formed an ad hoc research ethics support network (RESCOP). This collaborative network of support was instrumental in providing guidance to facilitate the rapid review of COVID-19 research (National Department of Health, 2015; World Health Organization, 2020c) and a repository of resources for RECs during COVID-19 (Rossouw et al., 2021). The importance of national and regional collaborative networks for RECs during public health emergencies has also been highlighted by Salamanca-Buentello et al. (2023) as part of their recommendations to strengthen the resiliency of RECs.

While rapid review processes for institutional-level RECs were generally implemented effectively, efforts to harmonise research ethics review did not work well during COVID-19. This may in part have been due to the absence of the NHREC for the critical periods of the first and second waves of COVID-19 in South Africa. Therefore, RECs were reliant on the South African National Department of Health’s Ethics in Research Guidelines (2015), which provide that “RECs may, at their own discretion, recognise prior review and approval of a research proposal by another registered REC to avoid duplication of effort” (National Department of Health, 2015: 43). However, participants were concerned about the consistency of reviews across different RECs and that a reciprocal review process might impact on the overall rigour of REC review. Factors that may impact on review consistency include experience in reviewing high-risk research and the level of rigour of research ethics review. Some RECs might also just generally be inclined to give favourable reviews. It is also possible that limited funding and resourcing of RECs might impact on review rigour.

Participants were concerned that standardising reciprocal review processes might result in potential gaps in ethical oversight and the protection of research participants. In addition, while some participants recognised the potential benefits of reciprocal review, the majority view was that lack of trust is deeply embedded in South Africa’s history. Moll (2021) suggests that many South Africans that have lived experiences of “biopolitical stratifications of life and how medicine has often worked alongside those stratifications” (Moll, 2021: 119). They also highlight that many South Africans come to COVID-19 with durable legacies and daily experiences of racism, including ongoing mistrust of medical and academic institutions and the enduring health disparities (Moll, 2021). In South Africa, knowledge of or direct experiences of exploitation and discrimination under Apartheid informs perceptions of research (Thabethe et al., 2018). Mistrust may also be linked to experiences of the broader socio-political context (Newman et al., 2011) of racial discrimination under Apartheid, and continued marginalisation of certain groups in post-Apartheid South Africa (Thabethe et al., 2018). Socioeconomic and political stability have been identified as key enabling factors for supporting robust REC systems and regulatory frameworks (Hummel et al., 2021; Rahimzadeh et al., 2023). The importance of building trust with research communities and other RECs was emphasised, and this might be facilitated through the development of a community of practice to enhance collaboration and knowledge exchange between RECs.

Further, Rahimzadeh et al. (2023) proposed incremental steps to build trust among RECs and provided practical recommendations, including making prior REC reviews available to other RECs considering reciprocity and making use of standardised review criteria to support the quality and consistency of REC reviews. However, wide implementation of reciprocal review remains theoretical, particularly during public health emergencies such as COVID-19. More work is needed to clearly establish the costs and benefits of mutual recognition of reviews (Rahimzadeh et al., 2023), compared to multiple reviews by single RECs for different research stakeholders, including researchers, RECs and research participants.

### Limitations and Strengths

This study is based in South Africa, and some findings, such as the absence of the NHREC for early stages of the pandemic, the development of a collaborative network of RECs during COVID-19 (RESCOP), and historical mistrust were unique to the South African context during COVID-19. We recognise that these contextual factors may limit the transferability of some findings but hope that we have provided sufficient thick description for these findings to be meaningful to researchers from other contexts. Our purposive sample of 21 participants also represents a small subset of the research ethics community in South Africa, but we feel potential selection bias was limited by the wide representation of RECs from different institutions across South Africa.

In addition, many RECs that were represented in this study were well-established, with most participants reporting more than 10 years of experience serving on their institutional REC. Approximately 50% of participants had formal postgraduate qualifications in ethics. Many RECs in South Africa are also highly experienced in reviewing clinical trials involving novel therapies or vaccine candidates for infectious diseases, due to the high disease burden of tuberculosis and HIV, and we therefore consider that the challenges of RECs’ pandemic preparedness and responses identified in this study are likely to be transferable to other national contexts.

We also acknowledge that not including REC administrators was a missed opportunity, particularly given the importance of operational challenges, changes in workload and ethics support systems that influenced REC functioning and coping during COVID-19. Future studies should include REC administrator’ views as they are essential stakeholders in building REC preparedness for future public health emergencies. We also recommend that researchers’ views of REC functioning during the COVID-19 pandemic should also be explored. Further, it is critical to explore how the workload and skills of RECs are recognised and valued by institutions, and to identify opportunities to improve the sustainability and resourcing of RECs.

## Conclusion

South African RECs faced significant challenges during the COVID-19 pandemic. Despite an initial sense of unpreparedness, RECs were able to adapt and maintain careful ethical oversight of both COVID-19 and non-COVID-19 research and the rigour of RECs reviews. Several important lessons for preparedness and response to future pandemics were identified. There is a need for heightened awareness among RECs of the ethical implications of publication pressures during public health emergencies. Training in research ethics, the responsible conduct of research, and research integrity must be prioritised to appropriately sensitise RECs, researchers and communities to essential competencies and requirements for ethical research during public health emergencies.

Strategies to enhance moral resilience of REC members are critical and should be implemented during interpandemic times. A key recommendation is the development of a community of practice to promote relationship-building and knowledge exchange between RECs. Incremental steps are needed to build trust and authentic partnerships among RECs in inter-pandemic times, to facilitate collaboration during future public health emergencies.

## Supplementary Material

Supplementary material

## Figures and Tables

**Table 1.** Descriptive characteristics of participants (*n* = 21).

Descriptive characteristics	*n*
REC status	
Chair	10
Member	11
Discipline	
Professional care, counselling, or health-related care	14
Laboratory or medical scientist	4
Law	1
Social Science	2
REC experience (years)	
1–5	4
6–10	3
11–15	6
>15	8
Ethics qualification	
None	10
Postgraduate diploma	6
Bachelor degree	1
Masters degree	1
Doctorate	3

**Table 2.** Three main themes and related sub-themes identified during qualitative analysis.

Themes	Sub-themes
Coping during COVID-19	Initially unprepared for COVID-19
	Strain of REC work
	Impact of national guidance absence
	Interconnectedness of pressures
Building REC capacity during pandemic times	Sense of making a meaningful contribution
	High level of commitment to REC functioning
	Emergence of innovative practices and collaboration
Consistently cautious approach to reciprocal review	Concerns for review consistency and competency Importance of trust
	Need to promote community building and knowledge exchange between RECs

## Data Availability

The datasets generated and analysed during the current study are not publicly available due to consent not being obtained for public sharing. However, data may be available from the corresponding author on reasonable request and with permission of the Stellenbosch University Health Research Ethics Committee and the University of Cape Town, Faculty of Health Sciences Human Research Ethics Committee.

## List of abbreviations

REC(s): Research Ethics Committee(s)
HCW(s): Healthcare workers
NHA: National Health Act 61 of 2003
NHREC: National Health Research Ethics Council
LMIC(s): Low- and middle-income countries
RESCOP: Research Ethics Support in COVID-19 Pandemic
