# Supplementary material for: Exploring views of South African research ethics committees on pandemic preparedness and response during COVID-19
Source: Res Ethics. Author manuscript; Available in PMC 2024 Dec 5. (PMC11619208; doi:10.1177/17470161241250274)
Supplement: Supplementary material [file NIHMS2029287-supplement-Supplementary_material.docx]

## Supplementary file: Interview Guide for REC Chairs/ Members

### Introductory (Descriptive) Questions

1. How long have you served on a research ethics committee (REC)? If Chair, how long have you served as Chair?
2. What expertise do you bring to the REC? (For example, area of specialisation, lay member, research ethics qualifications, qualitative or quantitative research knowledge/ experience).
3. Have you attended REC meetings regularly in the last six months?

### REC Preparedness and Response to COVID-19 (Including Meetings, SOPs and Review Processes)

1. What have been your experiences of REC meetings and processes during COVID-19?
2. Has anything changed from ‘regular’ meetings or any adaptation in review processes to:
   1. Prioritise national and multi-site ethical review;
   2. Facilitate COVID-specific review processes;
   3. Review novel, complex, adaptive trial designs;
   4. Facilitate or improve data safety and monitoring; and
   5. Facilitate or improve ethical oversight of approved COVID-19 research studies.
3. Have any changes in REC Standard Operating Procedures (SOPs) been made to expedite the review of COVID-19 research? In your experience, have there been any advantages or disadvantages associated with the change in SOPs; or alternatively, with keeping the SOPs the same?
4. In your experience, has engagement with researchers, regulators and other research stakeholders changed as a result of the COVID-19 pandemic and national lockdown? If yes, please explain.
5. In your experience, did the absence of the National Health Research Ethics Council impact on REC reviews and functioning during COVID-19 in 2020? Please explain why or why not.
6. Please give your perspective on how you feel the REC been coping with COVID-19 research and review processes? If you feel the REC is coping well, please give some examples to contextualise your answer or reasons why you think this. If you feel the REC has not been coping well, please give some examples to contextualise your answer or reasons why you think this.
7. In your experience, have there been any other effects of COVID-19 and national lockdown on REC review processes?

### Ethics Review of COVID-19 Research

1. Please tell me about your experiences of reviewing COVID-19 research; and the type of research (including level of risk) that you have reviewed.
2. In your experience, have there been any additional pressures or demands associated with reviewing COVID-19 research? (For example, pressure from researchers or institutions, national or international pressures, awareness of solidarity, public good of finding effective vaccine). If yes, do you perceive that these factors have influenced the REC review process in any way?

### Reciprocal Review and Harmonisation of SA REC Reviews

1. What is your position on mutual recognition of REC reviews/ approvals?
2. In your experience, has there been any formal or informal harmonisation of research ethic reviews or review criteria between RECs from different institutions in South Africa during COVID-19?
3. In your experience, has there been any formal or informal collaboration between RECs to minimise or avoid potential duplication of effort associated with multiple, independent, staggered REC reviews?
4. Are there any systems or processes in place for reciprocal review or mutual recognition of REC reviews/ approvals at your institution? In your experience, do these systems work or not?
5. In your opinion, what research ethics aspects can be subject to mutual recognition of REC review/ approval?
6. In your opinion, when is local REC review needed? Based on your experience, are there any aspects of the ethics review process that require local, contextualised REC review?
7. In your opinion, what factors might influence mutual recognition of REC review or reciprocal review? Prompts: does it make a difference if the REC of record and/ or researchers are local or international? Does the perception of equivalence of REC reviews play a role? Might the level or risk of vulnerability of participants play a role? Are there any other barriers or facilitators to mutual recognition of REC reviews or reciprocal review?
8. What is your perspective on how RECs might balance the potential risks and benefits of mutual recognition of REC reviews or reciprocal review; with the risks and benefit of multiple, independent, staggered REC reviews? In your opinion, how might this debate be moved forward?

### Ethical Issues associated with COVID-19 Research

1. What ethical, legal or health equity issues have you encountered in your review of COVID-19 research?
2. Specific prompts for perspectives on the following ethical issues:
3. Scientific validity
4. Social value
5. Fair selection of study participants
6. Risk: benefit
7. Community engagement, and respect for persons and communities
8. Informed consent
9. Collaborative partnerships
10. Health equity, equality and social justice issues
11. Storage and future use of COVID-19 data and/or sample
12. Social harms such as stigmatisation and discrimination
13. Structural socio-economic factors related to lockdown regulations and participant vulnerability
14. Placebo-controlled trials
15. COVID-19 vaccine research
